# Therapeutic advances in hemophilia: from molecular innovation to patient-centered global care

**DOI:** 10.3389/fmed.2025.1618464

**Published:** 2025-09-25

**Authors:** Zaure Dushimova, Marat Pashimov, Jamilya Kaibullayeva, Laura Danyarova, Elmira Kultanova, Gulnara Abdilova, Nagima Mustapayeva

**Affiliations:** ^1^Higher School of Medicine, Al-Farabi Kazakh National University, Almaty, Kazakhstan; ^2^JSC “Research Institute of Cardiology and Internal Diseases”, Almaty, Kazakhstan; ^3^JSC “Scientific Center of Pediatrics and Pediatric Surgery”, Almaty, Kazakhstan; ^4^Department of Nephrology, Asfendiyarov Kazakh National Medical University, Almaty, Kazakhstan

**Keywords:** hemophilia, non-replacement therapy, gene therapy, factor, immune tolerance induction

## Abstract

Hemophilia A and B are uncommon inherited bleeding disorders linked to the X chromosome, resulting from a lack of coagulation factors VIII or IX, respectively. Acknowledged for centuries, hemophilia was historically a dangerous condition lacking effective treatment methods. Significant progress in the 20th century brought about clotting factor alternatives and strategies for preventive treatment. This review offers a refreshed overview of both conventional and new therapies, such as gene therapy, by assessing their advantages, drawbacks, and potential future developments. The narrative review consolidates existing information regarding the pathophysiology of hemophilia, its classification, genotype–phenotype correlations, and advancements in treatment. It examines factor replacement therapies along with newer strategies like non-factor therapies, immune tolerance induction, and gene therapy, while evaluating how these treatments affect patient quality of life and worldwide access to healthcare. While factor replacement therapy is still essential, it entails regular infusions, substantial expenses, and potential risks of inhibitor formation. Innovations such as extended half-life treatments and subcutaneous therapies have enhanced adherence and lowered bleeding rates. Gene therapy has demonstrated the possibility of prolonged natural factor production but continues to encounter issues concerning long-term safety, durability, and accessibility. Newly emerging concerns include the underrepresentation of Hemophilia B in translational research, the immunological challenges associated with vector-based platforms, and significant global disparities in accessing advanced therapies, particularly in low-resource settings. In spite of progress, inequalities in treatment remain, with around 70% of patients globally unable to access crucial therapies.

## 1 Introduction

**Figure 1:**
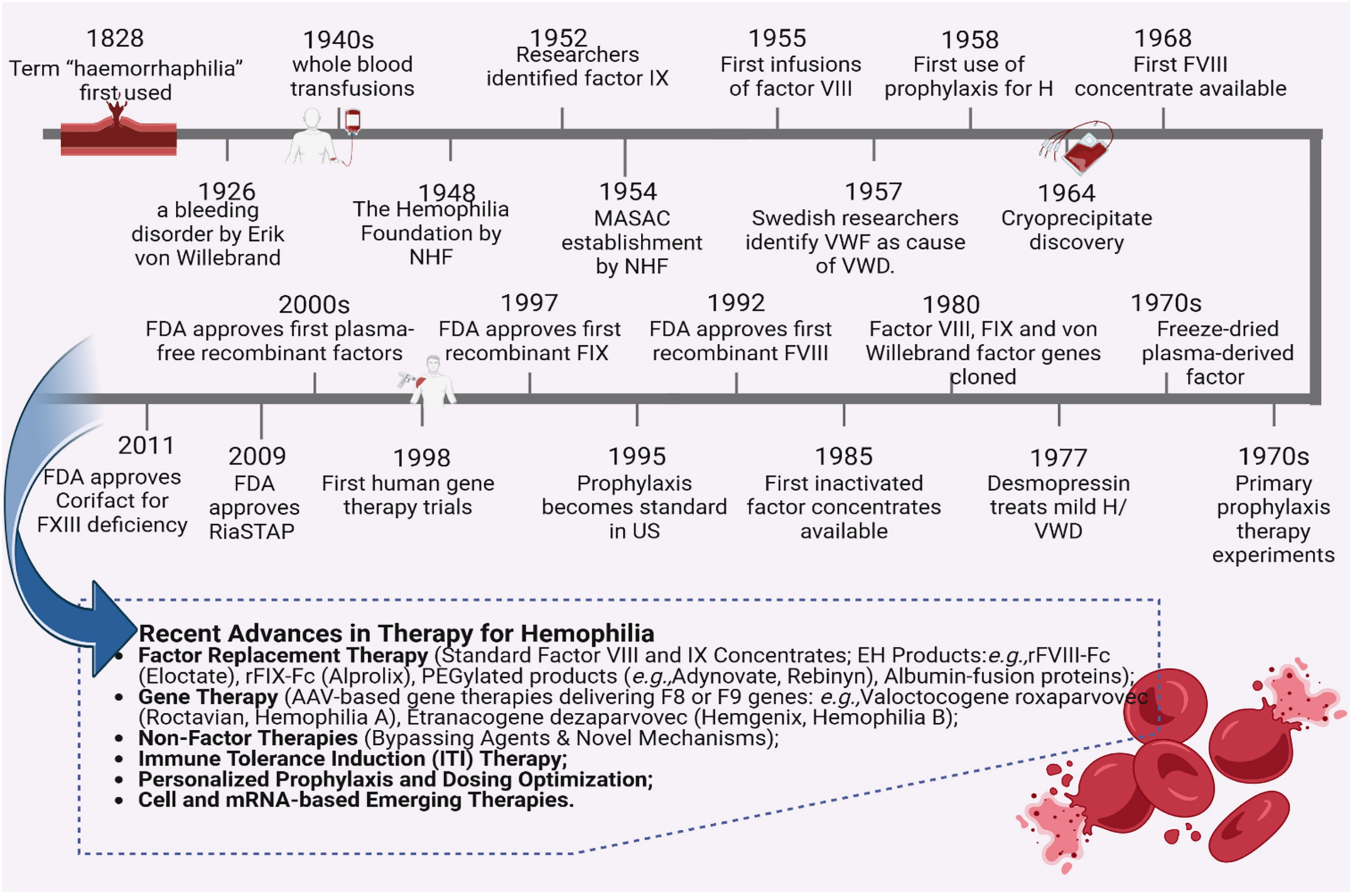
Historical timeline and therapeutic advances in hemophilia management. HA, Hemophilia A; VWD, von Willebrand disease; VWF, von Willebrand factor; FDA, U.S. Food and Drug Administration; NHF, National Hemophilia Foundation; MASAC, Medical and Scientific Advisory Council; FIX, Factor IX; FVIII, Factor VIII; FXIII, Factor XIII; U.S., United States.

Hemophilia has been acknowledged since antiquity, with evidence tracing back to Egyptian papyri and the Talmud in the 2nd century CE. The Talmud, a compendium of Jewish rabbinical literature, indicated that circumcision was not obligatory if two previous male siblings had succumbed post-procedure—an early signifier of hereditary bleeding disorders (1). In a similar vein, the New Testament narrates the account of a woman who suffered from incessant bleeding for a duration of 12 years until she attained healing through contact with the garment of Jesus (2). In the 10th century, the Arabian physician Abulcasis (Abu al-Qasim) documented instances of families wherein male individuals perished due to excessive hemorrhaging following minor injuries (3). By the 19th century, this ailment became colloquially known as the “royal disease” owing to its prevalence among European royal lineages (4). Notwithstanding these primordial observations, substantial scientific advancements in the comprehension and management of hemophilia did not materialize until the 20th century (5). Throughout this epoch, seminal breakthroughs—including the discovery of clotting factors, the formulation of replacement therapies, and the implementation of prophylactic interventions—effectively revolutionized the care of individuals with hemophilia. These progressions established a robust foundation for the innovations of the 21st century, which have dramatically transformed therapeutic approaches, as exemplified in recent advancements (Figure 1) (3, 4, 6, 7).

Hemophilia A (HA) and Hemophilia B (HB) are X-linked recessive blood clotting disorders resulting from a lack of coagulation factor VIII (FVIII, linked to the *F8* gene) or factor IX (FIX, associated with the *F9* gene), respectively (8). These elements are crucial in the intrinsic pathway of hemostasis, and their lack interferes with normal clot development, leading to extended bleeding episodes (9). Because the genetic foundation of HA and HB is located on the X chromosome, these disorders mainly impact males (XY), who do not have a second X chromosome that could contain a working gene version (10). Females (XX) usually act as carriers since they possess adequate levels of clotting factors from one functioning allele. Nonetheless, certain carriers might show symptoms due to lyonization, a mechanism in which one X chromosome is randomly inactivated in every somatic cell during early development (11). In instances of skewed or non-random X inactivation, the normal X may be selectively silenced, which decreases clotting factor production and causes a tendency to bleed (12). A frequent symptom in symptomatic female carriers is menorrhagia, which is heavy menstrual bleeding resulting from compromised hemostasis in the endometrial tissue (13). Although menorrhagia may arise from hormonal imbalances, uterine fibroids, or von Willebrand disease (VWD), its occurrence in hemophilia carriers necessitates assessment for clotting factor deficiencies (14–16). In healthy individuals, normal FVIII levels fall between 64 and 197 IU/dL, usually being lower in individuals with blood group O (55–150 IU/dL) than in non-O groups (71–186 IU/dL), attributed to reduced VWF levels that stabilize FVIII (17, 18). In hemophilia, severe cases have FVIII or FIX levels under 1 IU/dL, moderate cases range from 1 to 5 IU/dL, and mild forms are between 5 and 40 IU/dL, with the degree of bleeding related to factor concentrations (19). Uncontrolled bleeding, particularly into joints (hemarthrosis) and muscles, frequently happens in severe instances and can occur.

HB is frequently viewed as having a milder clinical presentation compared to HA, though this differentiation is still a topic of discussion. In HA, about 45–50% of severe instances result from an intron 22 inversion in the *F8* gene, a mutation that causes a total loss of gene function (20). Conversely, HB is mainly linked to missense mutations in the *F9* gene, representing approximately 55% of instances, whereas large deletions are rarer (~17%) (19). These genetic variations might affect phenotypic severity, as missense mutations usually lead to less severe bleeding tendencies. Additionally, in HA, missense mutations comprise about 75% of moderate cases and merely 16% of severe ones, indicating a link between mutation type and the severity of the disease (21). Furthermore, joint and muscle bleeds are the defining feature of hemophilia-related complications, frequently advancing to chronic arthropathy marked by cartilage deterioration, synovial swelling, and inflammation. Research indicates that nearly 90% of individuals with severe HA experience hemophilic arthropathy by the time they reach adulthood (22). This musculoskeletal injury results in decreased mobility and functional autonomy, greatly affecting quality of life, particularly in individuals with late or inadequate preventive care (23).

HA impacts roughly 1 in 5,000 to 10,000 male live births, making it one of the most prevalent inherited bleeding disorders worldwide (24). According to the World Federation of Hemophilia (WFH), an estimated 815,000 people around the globe have hemophilia, but only roughly 347,000 cases have been officially diagnosed, with around 276,900 classified as severe (25). Recent statistics from 2023 show that in Kazakhstan, there are 1,633 people affected by hemophilia, among them 533 are children. In reaction to the persistent disease burden, innovative treatment approaches have surfaced, such as long-acting factor concentrates, non-factor therapies like emicizumab, gene therapy, and new mRNA-based solutions. These advancements seek to enhance prophylaxis, decrease the frequency of bleeding, and improve patient quality of life by lessening treatment burden and providing more consistent hemostatic control (26). Nonetheless, obstacles persist. Elevated treatment expenses, restricted availability in low- and middle-income nations, inconsistent patient reactions, and the possibility of forming neutralizing antibodies (inhibitors) persist in limiting the complete clinical efficacy of these therapies. Additionally, although gene therapy presents the potential for a functional cure, long-term safety, expression durability, and immune response management remain subjects of ongoing research (27). These constraints emphasize the necessity for ongoing innovation and worldwide health strategies to guarantee fair access and sustainable treatment for everyone with hemophilia.

This review centers on the present state of hemophilia treatment, addressing both traditional and innovative therapeutic approaches. Conventional methods, like factor replacement therapy, have historically been the basis of treatment, yet they necessitate regular infusions and come with the possibility of producing neutralizing antibodies. To tackle these challenges, numerous contemporary alternatives have arisen. These consist of non-factor treatments, protocols for inducing immune tolerance, and gene therapy, which provides the possibility of sustained endogenous factor production. The review presents a brief summary of each of these approaches and emphasizes the therapeutic transition towards more effective, long-lasting, and less demanding treatments. Although gene therapy shows significant potential by addressing the fundamental genetic cause of hemophilia, obstacles like long-term effectiveness, immune reactions, and accessibility persist. Newly emerging concerns, including the underrepresentation of HB in translational research, the immunological complexity of vector-based systems, and global inequities in access to advanced therapies, are discussed to highlight future directions for more inclusive and equitable care. This review seeks to provide a current perspective on advancements in hemophilia care by examining these developments and addressing the continuous necessity to enhance treatment results for every patient.

## 2 Materials and methods

**Figure 2:**
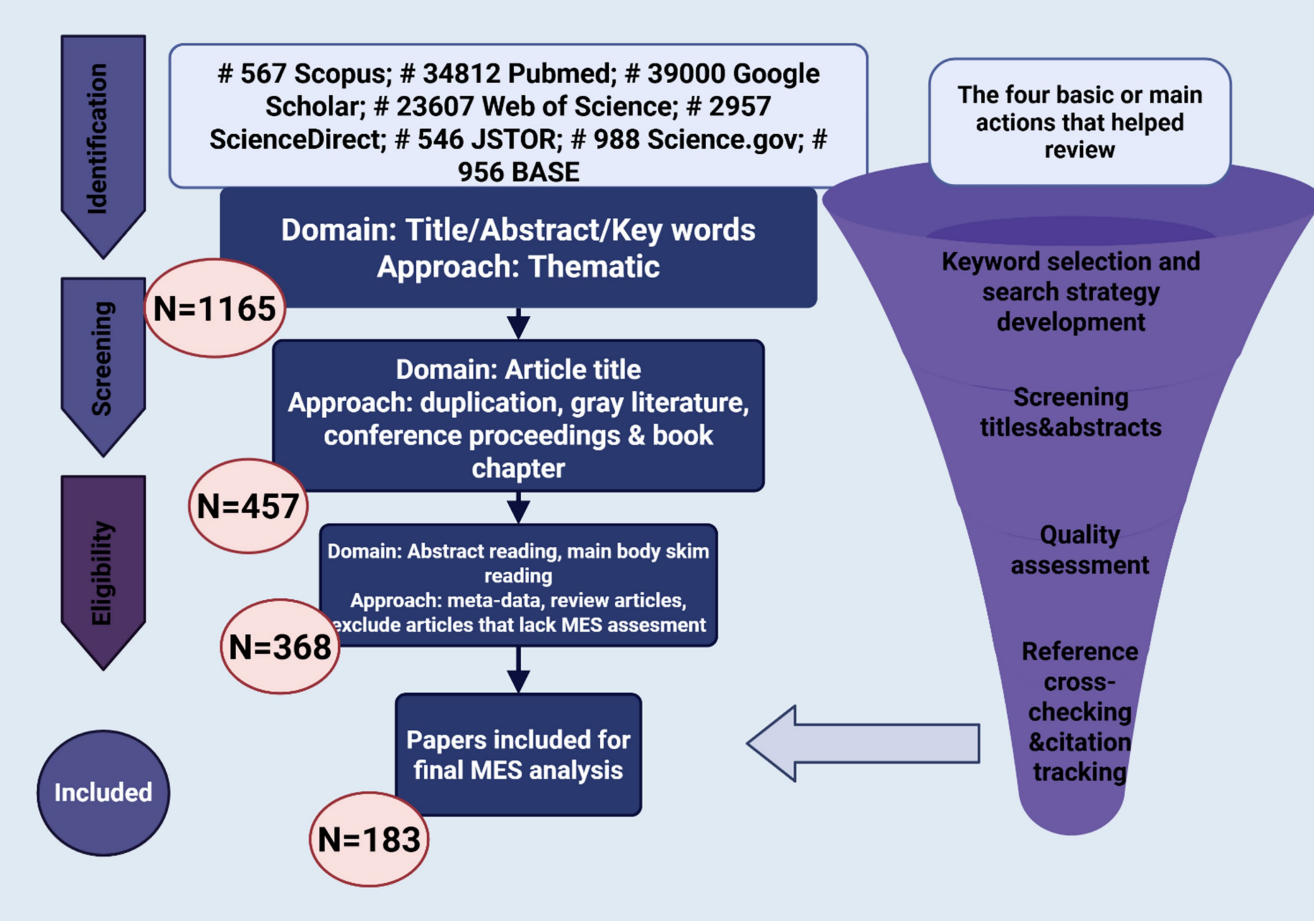
Systematic literature selection process.

A thorough literature review was performed to discover research concerning therapeutic improvements in hemophilia, emphasizing molecular advancements and patient-focused care. Investigations were conducted across seven databases, which included Scopus, PubMed, Google Scholar, Web of Science, ScienceDirect, JSTOR, Science.gov, and BASE (Figure 2). Following the application of keyword filters and the examination of titles and abstracts, 1,165 studies were initially chosen. After eliminating duplicates, grey literature, book chapters, and conference proceedings, 457 studies were left. Additional screening eliminated reviews and studies that did not directly focus on molecular or therapeutic aspects, leading to the selection of 183 articles for in-depth analysis.

## 3 Current therapies

Factor VIII and IX replacement therapies continue to be crucial in the management of hemophilia, backed by comprehensive clinical trials and real-world data that prove their effectiveness in managing bleeding episodes and minimizing long-term joint damage (15). Pioneering studies by Nilsson highlighted the significance of primary prophylaxis started before the age of two to avert hemophilic arthropathy (23), while secondary prophylaxis aims to stop additional joint damage once it has commenced (27). The advent of recombinant FVIII and later recombinant FIX enhanced treatment safety by decreasing dependence on plasma-derived products (28, 29). Nonetheless, the brief half-lives of traditional therapies, around 8–12 h for FVIII and 18–24 h for FIX, require regular infusions, creating adherence difficulties (19, 29, 30). Extended half-life (EHL) products, including rFIX-Fc fusion proteins and PEGylated rFVIII, have resolved this issue by permitting lengthier intervals between administrations (19). Even with these improvements, breakthrough bleeding and differences in individual pharmacokinetics underscore the necessity for tailored prophylaxis (31). Current hemophilia management includes four primary approaches: factor replacement therapy, gene therapy, non-factor therapies, and immune tolerance induction (ITI) (27, 32–34). Every method targets particular clinical requirements, with choices made according to severity, inhibitor status, patient preference, and availability of healthcare (34).

## 4 The replacement therapy

The development of replacement therapy for hemophilia started in 1948 with the transfusion of whole plasma, marking a significant advancement after identifying that hemophilia resulted from a lack of a plasma clotting factor (Table 1) (35). Sweden’s introduction of plasma-derived globulin fractions allowed for more focused treatment, albeit with restricted purity (36). A major breakthrough took place with the identification of cryoprecipitate, enabling FVIII-rich products to be derived from plasma of a single donor (37). Nonetheless, cryoprecipitate’s restricted FVIII content and the difficulties of storage and dosing rendered it an inadequate solution (38). Treatment for a 70 kg patient typically necessitated as many as 20 bags per dose, underscoring the demand for more effective formulations (39).

**Table 1:** Overview of factor replacement treatments for hemophilia.

Therapy type	Method of production	Major risks	Infection risk	Current use	Ref.
Used for HA (FVIII deficiency)					
Plasma infusion	Whole plasma infusion	Volume overload, allergic reactions	Moderate	Rare, emergency settings	(163)
Globulin fraction	Globulin fraction via Cohn’s method	Low purity, allergic reactions	Moderate	Obsolete	
Cryoprecipitate	Cryoprecipitation	Viral transmission (HBV, HCV, HIV)	High (esp. HCV, lower for HIV)	Limited, low-resource settings	
Early plasma-Derived FVIII Concentrates	Large pooled plasma; chromatography	High risk of viral transmission	Very high (up to 75% HIV in some cohorts)	Mostly replaced	(164)
Recombinant FVIII	CHO cell expression with human/animal proteins	Inhibitor formation, viral concerns	Low	Used in some countries	(46)
Recombinant FVIII (2nd/3rd Gen)	CHO cell expression without animal/human additives	Inhibitor formation	Low	Standard in many countries	(48)
Extended half-Life FVIII	PEGylation or Fc fusion	Unknown long-term effects	Low	Increasing use	(165)
Used for HB (FIX deficiency)					
Plasma infusion	Whole plasma infusion (contains FIX among all factors)	Volume overload, allergic reactions	Moderate	Used in low-resource settings	(164)
Prothrombin Complex Concentrates (PCCs)	Fractionation; contains FIX, II, VII, X	Thrombosis risk, imbalanced factor levels	Moderate	Used in many countries	(46)
Plasma-derived FIX concentrates	Plasma-derived FIX purification	Viral transmission (lower than early FVIII products)	Low	Increasing use in developed settings	(164)
Recombinant FIX (rFIX, 1st generation)	CHO cell expression (recombinant)	Inhibitor formation (rare), high cost	Low	Used in many countries	(47)
Extended half-life FIX (EHL-FIX)	PEGylation, Fc or albumin fusion	Unknown long-term safety, liver uptake concerns	Low	Increasing use in developed settings	(46)

CHO, Chinese Hamster Ovary (cells used in recombinant protein production); PEG, Polyethylene Glycol (used in PEGylation for extended half-life); Fc, Fragment crystallizable region (used in Fc fusion for extended half-life); AAV, Adeno-Associated Virus (vector used in gene therapy); HCV, Hepatitis C Virus; HBV, Hepatitis B Virus; HIV, Human Immunodeficiency Virus; IU, International Units; EHL, Extended Half-Life.

The pharmaceutical sector reacted by creating plasma-derived FVIII concentrates with enhanced specific activity using techniques like immunoaffinity chromatography (40). However, these concentrates, typically derived from extensive plasma pools, posed significant risks of viral transmission (39). In the 1980s, as many as 75% of individuals with severe HA acquired HIV from tainted commercial concentrates, and 20% of patients receiving single-donor cryoprecipitates were similarly impacted (40). In Italy, the HIV prevalence was significantly different between patients with HA and HB, probably because of variations in product usage. These incidents highlighted the necessity for enhanced viral safety (41).

By the late 20th century, considerable progress had been made to reduce these hazards (42). Donor screening, heat treatment, pasteurization, and solvent/detergent processing became standard practices in production methods (43). Nonetheless, these measures proved largely unsuccessful against non-enveloped viruses like parvovirus B19 and HA (44). Although contemporary recombinant products and extended half-life factor concentrates have mitigated many previous shortcomings, their expense and availability continue to be issues in low-resource environments (45).

The introduction of first-generation recombinant FVIII (rFVIII) concentrates represented a considerable advancement in the treatment of hemophilia, being produced using CHO cells modified to produce human FVIII cDNA in media rich in albumin (46, 47). Later technological advancements resulted in the creation of second-generation rFVIII products that omitted albumin from the culture process, along with third-generation products that eliminated it completely from both the culture medium and final formulation by utilizing synthetic stabilizers (48). These improvements increased product purity and reduced the risk of pathogen spread. At the same time, recombinant FIX (rFIX) was introduced for HB, providing a safer option compared to plasma-derived products. Additionally, to enhance rFVIII production, a variant lacking the B-domain was developed, resulting in improved yield and expression efficiency in cell lines. This molecular alteration has now emerged as the favored configuration in producing contemporary rFVIII treatments because of its stable pharmacokinetics and similar effectiveness (49–51). The development of recombinant factor therapies greatly enhanced the safety and scalability of hemophilia care, minimizing reliance on human plasma and the related viral hazards.

Despite the progress made by recombinant factor VIII (rFVIII) therapies, one of the major clinical hurdles in HA continues to be the formation of neutralizing antibodies, often known as inhibitors. These antibodies can greatly hinder or nullify the effectiveness of replacement therapy. The occurrence of inhibitors is consistently found to be greater in patients receiving rFVIII than in those treated with plasma-derived FVIII (pdFVIII) (32). Historical research suggested that inhibitor development occurred in about 24% of pdFVIII recipients compared to 33–52% of those using rFVIII (52). Regulatory trial results have indicated comparable increased rates—between 29.7 and 38.0%—for products such as Kogenate®, Recombinate®, and ReFacto® (53). Recent findings from the SIPPET trial further supported these concerns, indicating a cumulative incidence of inhibitors at 44.5% with rFVIII versus 26.8% with pdFVIII, resulting in a hazard ratio of 1.87 (54). Nonetheless, recent real-world evaluations and prospective research have yielded mixed outcomes, with certain 2020–2023 groups indicating similar immunogenicity for rFVIII and pdFVIII, probably affected by genetic variations, treatment intensity, and age at initial exposure (55). Conversely, the development of inhibitors in HB is uncommon, with incidence rates approximately 3% for both plasma-derived and recombinant FIX concentrates (56). However, these inhibitors can cause serious anaphylactic reactions in rare instances, requiring careful observation. Due to the clinical effects of inhibitors, prioritizing their prevention and early detection remains essential in improving hemophilia care.

Even slight decreases in baseline factor VIII or IX levels are clinically important since bleeding occurrence is closely linked to remaining clotting activity. Individuals with initial factor levels of 5 IU/dL generally undergo one joint bleed each year, whereas those with levels nearer to 3 IU/dL might have two or more occurrences annually (57). Significantly, just one joint bleed can activate the pathophysiological processes that lead to hemophilic arthropathy. Repeated hemarthroses result in persistent synovitis, iron accumulation, cartilage breakdown, and gradual bone erosion, leading to joint deformity, stiffness, and loss of muscle mass (58). Recent imaging and biomarker research has confirmed that subclinical bleeds, which are not detectable through standard evaluation, may also contribute to early joint damage, especially in patients receiving inadequate or delayed prophylaxis (58, 59). These results highlight the significance of timely, personalized preventive treatment approaches that focus on not just avoiding severe bleeding but also on maintaining long-term musculoskeletal well-being.

In recent decades, products of rFVIII have seen major improvements in purity, safety, and production techniques. The initial versions were made using proteins from animals and human serum albumin, which both carried risks for immune reactions (23). To tackle these issues, second-generation rFVIII formulations removed animal proteins from production, while third-generation products entirely eliminated both animal and human components by using synthetic stabilizers (50). These enhancements have significantly decreased the likelihood of negative immune responses and the spread of blood-borne pathogens. Another line of development has aimed at prolonging the half-life of replacement therapies. Methods like PEGylation and fusion with the Fc segment of immunoglobulins have been utilized on recombinant clotting factors, leading to prolonged circulation duration. For instance, the half-life of rFVIII has been elevated to about 19 h, whereas rFIX formulations now reach half-lives of as much as 100 h (30, 31, 60, 61). These improvements have enhanced treatment compliance and accessibility, especially for pediatric and elderly patients who struggle with regular infusions.

Nonetheless, in spite of these developments, the steep price of recombinant and extended half-life products restricts their availability in low- and middle-income nations. Consequently, numerous patients in these areas still rely on plasma-derived concentrates, which are not as effective and pose a greater risk of complications. Tackling this gap necessitates a worldwide initiative to increase access, mitigate the financial strain of treatment, and strengthen health system capabilities. The future of hemophilia treatment relies not only on ongoing technological advancements but also on health policy changes that ensure sustainable, equitable access to replacement therapy globally (25, 60).

## 5 Recent developments in non-replacement therapy

In recent years, the treatment options for hemophilia have broadened from conventional factor replacement therapies to encompass non-replacement approaches, many of which utilize monoclonal antibodies. This change is motivated by the necessity to address the challenges linked to replacement therapy, such as the regular intravenous infusions and the emergence of neutralizing inhibitors in a significant percentage of patients with HA (53, 55). Antibody-driven strategies present hopeful options by replicating or boosting natural coagulation processes without requiring the substitution of the absent factor. For instance, bispecific antibodies like emicizumab can effectively replace FVIII by binding to activated FIX and FX at the same time, which aids in the formation of the tenase complex (33). Additionally, antibodies have been engineered to aim at natural anticoagulants like tissue factor pathway inhibitor (TFPI), or to inhibit regulatory proteins using small interfering RNA (siRNA) strategies, as shown by treatments such as fitusiran, which lowers antithrombin production (62). These advancements greatly decrease the frequency of administration and remove the requirement for venous access, thereby enhancing adherence and quality of life. Nonetheless, although they offer benefits, worries persist about long-term safety, particularly thrombotic risks, and their high price continues to restrict access in low-resource environments (25).

Emicizumab is a bispecific, humanized monoclonal antibody engineered to replicate the cofactor function of factor VIII by binding to activated factor IX (FIXa) and factor X (FX) at the same time, thus aiding in the transformation of FX to FXa and enhancing subsequent thrombin production (63). In contrast to endogenous FVIII, which activates solely during coagulation, emicizumab stays active in circulation and works independently of FVIII levels, ensuring its effectiveness even with FVIII inhibitors present (33). It was created by selecting from thousands of candidate antibodies for ideal pharmacologic qualities, such as strong binding affinity, the ability for subcutaneous delivery, and an extended half-life of about 30 days (64). This permits adaptable dosing schedules, varying from once a week to every 4 weeks. Clinical trials, especially the crucial HAVEN 1–4 studies, have shown emicizumab’s effectiveness and safety in HA patients, regardless of inhibitor presence, across multiple age groups. Later research, such as HAVEN 7, has broadened its assessment to include infants and additional conditions like acquired hemophilia and VWD (65). Emicizumab notably lowered yearly bleeding rates compared to standard FVIII prophylaxis and is linked to enhanced treatment adherence owing to its less frequent administration and user-friendliness. Nevertheless, uncommon negative events like thrombotic microangiopathy (TMA) and thrombosis have been noted, mainly in patients who were given high amounts of activated prothrombin complex concentrate (aPCC) at the same time, particularly those exceeding 100 U/kg/day for over a day. Nevertheless, its immunogenicity characteristics are similar to those of other therapeutic monoclonal antibodies, exhibiting a low rate of anti-drug antibodies. The effectiveness of emicizumab has led to a transformation in HA treatment, progressively substituting conventional FVIII concentrates in various nations owing to its safety, efficacy, and lower treatment demands (33, 63).

Mim8, created by Novo Nordisk (Måløv, Denmark), stands for a new class of bispecific antibodies intended for the treatment of hemophilia A (HA) (66). With the Duobody platform, Mim8 aims at FIX and FX in its solution and operates similarly to emicizumab by connecting these two coagulation factors (67). In contrast to emicizumab, Mim8 attaches to different epitopes on FIXa and FX, which may provide unique therapeutic benefits (68). It is designed for subcutaneous use and includes enhanced membrane activation, low immunogenic potential, and decreased viscosity. Initial laboratory research validated its capacity to reinstate thrombin generation, while current phase 1 and 2 clinical trials are assessing its safety and effectiveness (69). MG1113, developed by Greencross (Gyeonggi-do, Korea), is an IgG4 monoclonal antibody that targets the Kunitz domain 2 of TFPI, demonstrating encouraging preclinical results in boosting thrombin production and minimizing bleeding in hemophilia models (70, 71). Befovacimab (BAY 1093884) developed by Bayer targets Kunitz domains 1 and 2 of TFPI and initially showed effective bleeding management, but subsequent worries regarding thrombotic risk emphasized the necessity for additional safety assessment (72). Marstacimab, created by Pfizer, focuses on the Kunitz-2 domain of the TF/FVIIa complex and has demonstrated encouraging decreases in bleeding rates during preliminary clinical trials (73).

Concizumab is a humanized IgG4 monoclonal antibody that specifically binds to the Kunitz-2 domain of TFPI, which promotes thrombin production by influencing the extrinsic coagulation pathway. Originally created for individuals with HA and HB who have inhibitors, the medication showed considerable effectiveness in clinical studies. A phase 3 trial showed a significant decrease in annualized bleeding rates, recording only one bleeding incident in the concizumab group versus 24 occurrences in the placebo group (74). In 2023, concizumab was granted regulatory approval in Canada for HB treatment in patients older than 12 years, particularly in those with factor IX inhibitors. It was later authorized for use in Australia and Switzerland, while evaluations by regulators are still in progress in the United States, the European Union, and Japan (75). In contrast to standard replacement therapies, concizumab provides the benefit of subcutaneous delivery and a positive bleeding profile, potentially enhancing adherence and the quality of life for patients. Nonetheless, ongoing monitoring is essential because of the possible thrombotic risk seen in patients with comorbid conditions or those using bypassing agents simultaneously. As a symbol of the expanding category of non-factor therapies, concizumab highlights the transition to targeted, antibody-driven methods in hemophilia treatment (76).

## 6 Advances in cellular therapy

Cell therapies for hemophilia require the genetic alteration of several cell types, such as endothelial progenitor cells, fibroblasts, adipocytes, hepatocytes, and stem cells, to produce coagulation factors outside the body before being transplanted into patients. This treatment approach has demonstrated potential in tackling coagulation deficiencies; nonetheless, a significant challenge is ensuring the prolonged expression of clotting factors by the transplanted cells over time (77–95). A clinical trial carried out by Roth et al. (77) was the first to indicate a statistically significant enhancement in blood coagulation after cell therapy. In this study, six individuals with severe HA were administered between 1 and 4 × 10^8^ fibroblasts that were genetically altered via a plasmid to produce FVIII. Among those who were given the highest dosage, FVIII activity rose by about 1 to 5 percent. Even with these positive outcomes, the therapeutic impact was short-lived owing to insufficient long-term factor expression.

Later studies have concentrated on utilizing retroviral and lentiviral vectors (LVs) to modify hematopoietic stem cells so they can express FVIII or FIX. These methods have produced enduring rises in coagulation factor levels, though findings have only been shown in animal studies (82–84). Further research has investigated the utilization of circulating blood cells like platelets and red blood cells as alternative carriers for the expression of clotting factors (85–87). Results show that FVIII expressed ectopically in platelets of transgenic mice seems to be immune to circulating inhibitors, suggesting it may serve as an effective therapy for HA (88, 89). Omori and associates detailed the alteration of hematopoietic stem cells via LVs guided by platelet- or megakaryocyte-specific promoters, subsequently transplanted into recipient mice (80, 81). This approach resulted in notable decreases in bleeding occurrences. Additionally, promoting platelet-targeted expression of FVIII or activated FVII has been demonstrated to reduce the incidence of bleeding in hemophilia mouse models (82, 90, 91). An additional approach entailed breeding experimental animals with transgenic mice that produced FVIII regulated by the endothelial-specific Tie-2 promoter, which also increased FVIII activity and minimized bleeding risks (92).

In the past few years, advancements have been made in employing induced pluripotent stem cells (iPSCs) for treating hemophilia. This method is especially appealing since the population of cells that produce coagulation factors is generally more stable (78). Xu et al. were the pioneers in showing the therapeutic application of iPSCs in models of hemophilia (78). In a pertinent study, Kashiwakura et al. (79) demonstrated that iPSCs, modified with a lentiviral vector and differentiated into endothelial cells, markedly alleviated bleeding symptoms in HA mice after intraportal injection. Nonetheless, maintaining prolonged gene expression and cell survival after transplantation continues to be a challenge. To overcome this constraint, scientists have utilized 3D scaffold technology and cell sheet engineering (93, 94). These methods have facilitated extended gene expression and enhanced the survival of transplanted cells. For instance, the implantation of a cell sheet that produced FVIII in hemophilic mice resulted in consistent expression of the clotting factor for almost 1 year, marking a notable progress in the creation of enduring cell-based treatments (95).

## 7 Gene therapy: from molecular discovery to clinical implementation

Gene therapy is rising as an attractive curative alternative for hemophilia by facilitating the internal production of FVIII or FIX via the transfer of a functional gene copy (96). Hemophilia is especially appropriate for gene therapy because its condition results from a lack of a single protein that exists in limited amounts, so even slight rises in factor levels are of clinical significance (97). Clinical experience and natural history research have reliably demonstrated that keeping clotting factor activity over 5% of normal can significantly reduce bleeding risks (98). In contrast to several monogenic disorders, hemophilia treatment does not require strict regulation of transgene expression, since there is a broad therapeutic range for circulating FVIII and FIX (99). Additionally, precise monitoring is achievable through standard coagulation tests that are commonly accessible in laboratories worldwide (100). Preclinical animal models, such as knockout mice and dogs with HA or HB, have offered important insights into the effectiveness of gene transfer and aided in refining delivery strategies (101).

### 7.1 Adeno-associated virus vectors (AAV)

AAV vectors continue to be the top choice for gene delivery in hemophilia because of their advantageous safety and transduction efficacy. AAVs are parvoviruses that cannot replicate on their own and need a helper virus for effective infection, while recombinant variants remove the wild-type viral coding regions, thus lowering immunogenicity (102). Multiple AAV-based gene therapies, including Luxturna, Zolgensma, and Glybera, have gained regulatory approvals for various monogenic disorders, which underscores their clinical efficacy (103). Although initial studies focused on AAV2, subsequent research has identified a broad spectrum of serotypes, each exhibiting distinct tissue tropism and immunological profiles suitable for diverse therapeutic applications (104). These vectors can aim at both dividing and non-dividing cells, such as hepatocytes, which are suitable for gene expression in hemophilia (105). Improvements in vector design, such as tissue-specific promoters and codon optimization, have greatly boosted protein expression levels (106). Codon optimization utilizes favored codons found in highly expressed liver genes, like albumin, to enhance translation efficiency (107). Furthermore, capsid modification has facilitated the creation of synthetic AAVs exhibiting improved potency, tissue specificity, and broader packaging abilities. Nonetheless, AAV vectors have a restricted packaging capacity of about 5 kilobases, which complicates the delivery of larger genes such as *F8*. To address this, approaches like truncated yet functional transgene variants have been created to comply with AAV constraints while maintaining therapeutic effectiveness (108).

A highly effective and commonly used method for introducing therapeutic genes into somatic cells is viral vector-mediated transduction. Among these vectors, AAVs are preferred for gene therapy for various monogenic diseases, such as hemophilia (108). So far, multiple AAV-based treatments have received regulatory approval for a range of conditions: Lumevoq (Leber Hereditary Optic Neuropathy), Luxturna (Leber congenital amaurosis), Zolgensma (spinal muscular atrophy), Glybera (lipoprotein lipase deficiency), ROCTAVIAN (HA), Hemgenix and BEQVEZ (HB), and Elevidys (Duchenne muscular dystrophy) (109–111). These vectors are regarded as safe because they are non-pathogenic to humans, have a low immunogenic profile, and cannot replicate on their own without the assistance of a helper virus like adenovirus or herpesvirus. Recombinant AAVs omit wild-type coding sequences, thereby decreasing the likelihood of cell-mediated immune reactions. Initially, AAV was the main concentration in early gene therapy studies, but now over 100 naturally occurring serotypes have been identified, each possessing distinct tissue tropism and immune characteristics. This variety enables precise transduction of both proliferating and non-proliferating cells. Additionally, the genetic adaptability of AAVs permits enhancement via powerful tissue-specific promoters and codon optimization methods, like employing sequences that are preferentially expressed by highly active liver genes such as albumin. Bioengineered AAV capsids have been created to enhance packaging capacity, tropism, and effectiveness. Nonetheless, AAV vectors possess a restricted genome capacity (~4.7–5 kb), requiring alternative approaches such as employing shortened but operational gene constructs (110, 111).

Due to the FIX gene’s smaller size and less complex processing needs (~1.5 kb), HB was the initial target for AAV-driven gene therapy. Innovative research by Katherine High’s team showed that therapeutic FIX expression occurs after administering an AAV2 vector into the hepatic artery. Despite FIX levels rising temporarily to approximately 10%, an accompanying asymptomatic increase in transaminase levels followed by a reduction in transgene expression indicated immune-mediated damage to transduced hepatocytes (110, 112). Importantly, in primate models, FIX levels stayed consistent even with doses ten times greater, reinforcing the safety of hepatic AAV administration.

Unlike AAV2, which generates antibodies in approximately 70% of humans, AAV8 shows a lower seroprevalence of around 25% and has a strong preference for the liver. This serotype was utilized in a groundbreaking research project by St Jude and University College London, in which enduring FIX expression was obtained through systemic delivery. These discoveries established a foundation for future constructs like AMT-061 (etranacogene dezaparvovec), which integrates the gain-of-function Padua variant of FIX and employs an AAV5 vector. In the HOPE-B phase 3 trial (NCT03569891), 54 patients administered a single intravenous dose of 13 × 10^13 vg/kg attained an average FIX activity of 36.9% after 18 months, with a 64% reduction in bleeding events and a 97% decrease in factor use, despite having pre-existing anti-AAV5 antibodies (112).

Furthermore, AMT-061 does not require immunosuppressive treatment, in contrast to previous candidates. In three individuals, FIX activity increased to 47% over 26 weeks without any elevation in liver enzymes (Table 2) (113). This differs from constructs such as SPK-9001 (fidanacogene elaparvovec), which employs a bioengineered AAV and FIX-R338L transgene with lower CpG content. While SPK-9001 achieved consistent FIX levels of 22.9% and decreased ABR to 0.4, an abundance of CpG motifs has been associated with Toll-like receptor 9 activation and corticosteroid-resistant transaminitis (114).

**Table 2:** Key clinical trials and outcomes of AAV-mediated gene therapies in hemophilia.

Study/trial name	Therapy	Target	Vector/serotype	Key findings	Adverse events	Clinical phase	Ref.
Initial FIX Study	AAV2-FIX	HB	AAV2	Increased FIX to ~10%, then declined	Transient ALT elevation	Pilot study	(111)
UCL	AAV8-FIX	HB	AAV8	Sustained FIX expression, improved transduction	Reduced immunoresistance (~25%)	Preclinical/early clinical	(112)
AMT-061/HOPE-B	Etranacogene	HB	AAV5	Mean FIX activity 36.9%, ↓64% bleeding, ↓97% FIX use	No corticosteroids needed	Phase 3	(113)
SPK-9001	Fidanacogene	HB	Engineered AAV	FIX activity 22.9%, ABR ↓ from 8.9 to 0.4	CpG-induced transaminitis	Phase 1/2	(166)
BBM-H901	Codon-optimized FIX	HB	Modified liver-tropic AAV	FIX ~36.9% at 1 yr.	ALT/aspartate elevation in 20%	Phase 1	(115)
BMN 270 (BioMarin)	Codon-optimized FVIII	HA	AAV (B-domain)	Mean FVIII ↑ to 41.9 IU/dL	ALT increase	Phase 3	(116)
SPK-8011	Codon-optimized FVIII	HA	Bioengineered LK03 capsid	16/18 maintained FVIII expression	ALT-related expression loss in 2	Phase 3	(117)

“↑” Signifies an increase (for instance, in FVIII or FIX activity), whereas “↓” represents a decrease (such as in bleeding rate or factor consumption). The symbol “~” signifies approximately.

In China, the BBM-H901 vector, which employs a specially designed liver-targeted AAV capsid along with a codon-optimized Padua FIX gene, attained FIX levels of 36.9% 1 year post-administration at a dosage of 5 × 10^12^ vg/kg; however, 20% of the patients exhibited increased transaminase levels (115). These findings validate that achieving stable, near-physiological FIX levels is feasible, a goal that was previously considered unachievable. Nonetheless, despite these levels representing a significant therapeutic advancement, the sustainability of gene expression beyond the initial year remains uncertain, underscoring the necessity for extensive long-term follow-up studies to ascertain whether hepatocellular expression maintains or diminishes due to immune-mediated clearance or vector silencing. Advancements have also been observed in gene therapy for hemophilia A (HA), notwithstanding the inherent difficulties associated with the extensive FVIII coding sequence and its naturally reduced expression efficiency. BioMarin’s BMN 270, which integrates B domain deletion and codon optimization, reached FVIII levels of 41.9 IU/dL in a phase 3 trial that included 134 patients (76, 116). Spark Therapeutics has developed SPK-8011 utilizing an altered LK03 capsid, facilitating sustained FVIII expression in 16 out of 18 patients for durations up to 47.6 months (117). Nevertheless, a considerable number of patients required immunosuppressive therapy to manage elevated liver enzymes, indicating that transaminitis may restrict widespread utility, particularly in resource-constrained environments where regular monitoring and intervention are less practical. Transaminitis persists as a frequent complication across various AAV serotypes and production methodologies (64, 116–120), while the rare integrations of AAV2 near oncogenes have prompted theoretical safety apprehensions (107, 121). These observations highlight that while contemporary gene therapy vectors exhibit clinical effectiveness, the design of vectors and delivery systems necessitates further optimization to alleviate hepatotoxicity and the oncogenic risks associated with integration. Furthermore, the criteria for patient selection, particularly concerning pre-existing liver conditions and neutralizing antibodies, should be refined to ensure both safety and therapeutic efficacy.

The presence of neutralizing antibodies (NAbs) against AAV capsids, identified in 20–70% of individuals, presents a considerable hurdle for the eligibility of gene therapy. While strategies such as serotype switching and the utilization of modified capsids, including AAV17, have shown promise in preclinical studies, the issue of human cross-reactivity limits their clinical applicability. Physiological factors, such as uptake by liver sinusoidal endothelial cells and Kupffer cells, further hinder the transduction efficiency of hepatocytes by filtering out particles smaller than 0.23 μm, emphasizing the urgent need for approaches that circumvent hepatic clearance (106). These biological impediments indicate that merely engineering novel capsids may be insufficient unless combined with tactics to evade immune recognition and reticuloendothelial system absorption, including transient immunosuppression, encapsulation of capsids with polymers, or targeting alternative tissue types. Recent progress has led to the regulatory endorsement of therapies like fidanacogene elaparvovec (Beqvez) for adult patients with HB and low baseline FIX activity who lack NAbs to AAVRh74var (116–119). In HA, investigational therapies such as valoctocogene roxaparvovec, SPK-8011, and SB-525 have significantly reduced bleeding incidents and improved FVIII activity through the application of optimized vectors and promoters (106, 122). Nevertheless, the longevity of expression in certain instances remains variable, particularly due to the phenomenon of vector epigenetic silencing, indicating a requirement for further optimization of vector genomes and promoter constructs. Innovations in vector design and production methodologies, despite challenges associated with baculovirus systems, continue to impact the discipline (Table 3) (59, 106, 122–125). However, the scalability and consistency of AAV manufacturing from batch to batch persist as significant obstacles, raising issues regarding reproducibility, cost, and global accessibility, especially in low- and middle-income nations. Future advancements will hinge not only on enhancing clinical efficacy but also on the establishment of cost-effective and scalable delivery mechanisms to guarantee equitable access.

**Table 3:** AAV-based studies for HA (59, 106, 122–125).

Name	Company	Clinical phase	Vector	Key findings	Adverse events	Trial ID	Ref.
BBM-H803	Belief BioMed	Phase 1	AAV	FVIII expressed long term from liver cells.	Not specified	NCT05454774	(106)
ASC618	ASC Therapeutics, Milpitas, CA, USA	Phase 2	AAV8	Encodes B domain of codon-optimized human FVIII with liver-specific promoter.	Not specified	NCT04676048	(106, 123)
PF-07055480	Pfizer (formerly Sangamo Therapeutics)	Phase 2	AAV2/6	Shorter *F8* expression duration, high liver tropism.	Mild AST, ALT increase, fever, hypotension.	NCT03061201	(124)
SPK-8011/RG6357	Roche, Basel, Switzerland (with Spark Therapeutics)	Phase 2	AAV-LK03	12.6 ± 7.3% FVIII maintained at 26–52 weeks.	2 patients lost FVIII expression.	NCT03432520	(125)
ANB-010	BIOCAD, Saint Petersburg, Russia	Phase 2	AAV	Encodes human FVIII; study approved for clinical trial in 2023.	Not specified	ANB-010-1/EDELWEISS	(59)
SB-525	Pfizer (formerly Sangamo Biosciences)	Phase 3	AAV2/6	↑FVIII activity in cohort 3 with no bleeds after 3 weeks.	ALT, AST elevated.	NCT04370054	(106)
BMN 270 (ROCTAVIAN™)	BioMarin Pharmaceutical, Novato, CA, USA	Phase 3	AAV5	↓ABR, ↑FVIII, ↓treatment usage; EMA & FDA approval.	ALT increase, mild transaminitis.	NCT03061201	(106)

AAV-mediated gene therapy for HB has advanced considerably, with a multitude of clinical investigations illustrating its effectiveness and long-lasting benefits (Table 4). Preliminary research utilizing AAV2 and AAV8 serotypes demonstrated that hepatic-targeted gene delivery could safely enhance FIX activity to therapeutic thresholds (126). These foundational findings corroborated the practicality of employing adeno-associated viral vectors to attain sustained FIX expression following a singular intravenous administration (127). Subsequent investigations have incorporated more sophisticated vectors, such as AMT-061, which employs the highly effective Padua FIX variant alongside an AAV5 capsid. In the HOPE-B phase 3 trial, this intervention achieved an average FIX activity of 36.9% and resulted in a 64% reduction in hemorrhagic episodes as well as a 97% decline in the necessity for FIX replacement therapy, even amongst patients possessing pre-existing AAV5 antibodies (128). Additional therapies, including SPK-9001, BBM-H901, AskBio009, and PF-06838435 (Beqvez), have reported positive outcomes, with Beqvez recently receiving regulatory endorsement in Canada and the United States (59, 129–132). Nevertheless, in spite of these advancements, variability in individual patient responses persists as a significant limitation, with certain subjects exhibiting lower-than-anticipated transgene expression or encountering transient elevations in hepatic enzymes necessitating corticosteroid intervention. This variability accentuates the intricate interplay among vector design, host immune characteristics, and hepatic health, emphasizing the necessity for personalized pre-treatment screening methodologies to more accurately forecast outcomes. Furthermore, the long-term persistence of FIX expression beyond a five-year period remains uncertain, as follow-up data are still insufficient, prompting inquiries regarding the potential requirement for re-administration or adjunctive therapies in the future (59, 115, 126, 127). Another pressing issue is the current financial burden of approved gene therapies such as Beqvez, which surpasses $3 million per dose, engendering considerable challenges concerning accessibility and reimbursement within both public and private healthcare frameworks (132). Moving forward, addressing these clinical, immunological, and economic obstacles will be essential to ensure that AAV-mediated gene therapies can realize widespread and equitable benefits for the hemophilia B patient demographic.

**Table 4:** AAV-based studies for HB.

Name	Company	Clinical phase	Vector	Key findings	Adverse events	Trial ID	Ref.
BBM-H901	Belief BioMed	Phase 1	Modified AAV vector delivering codon-optimized FIX Padua gene	Mean FIX activity 27.5% at 15 months, 64.5% had no bleeds	None in year 1, steroids given in some patients	NCT04135390	(59, 115)
VGB-R04	Shanghai Vitalgen BioPharma Co	Phase 1/2	Novel AAV vector with high-activity FIX variant	Median ABR reduced from 12 to 0, target joints from 1.5 to 0	20% had ALT/aspartate aminotransferase increase	Not yet registered	(126)
ANB-002	BIOCAD	Phase 2	BIOCAD proprietary AAV vector delivering human FIX gene	Trial authorized by Russian Ministry of Health in 2023	Not reported yet	ANB-002-1/SAFRAN	(127)
Idanacogene elaparvovec	Pfizer/Spark Therapeutics	Phase 2	AAV vector delivering FIX Padua gene	Mean FIX activity 27.5% at 15 months, 64.5% had no bleeds	None in year 1, steroids given in some patients	NCT02484092	(128)
AskBio009	Takeda	Phase 2	AAV2-derived vector with liver-specific promoter for FIX Padua	Enables hyperactive FIX (R338L) delivery using AAV2	Not specified	NCT01687608	(130)
AMT-061 (etranacogene dezaparvovec)	uniQure/CSL Behring	Phase 3	AAV5 vector delivering LP1-driven FIX Padua gene	96% reached sustained FIX levels, 36.9% mean FIX activity	ALT elevation in 21, 17% used corticosteroids	NCT03569891	(131)
PF-06838435 (Beqvez)	Pfizer	Phase 3	AAV-Spark100 vector encoding FIX Padua	71% reduction in bleeding, 92% reduction in factor use	Mild, 62.2% required corticosteroids for 107 days avg	NCT03861273	(132)

### 7.2 Lentiviral vector-based approaches in hemophilia

LVs have become a promising option in gene therapy, especially for diseases like hemophilia. In contrast to AAVs, which usually keep transgene expression episomally, LVs incorporate their genetic content into the host genome, guaranteeing stable and extended expression in dividing cells, a vital benefit for long-lasting therapeutic results. While worries about insertional mutagenesis are present, improvements in self-inactivating LVs have significantly lowered this danger (133, 134). LVs demonstrate low immunogenicity, which facilitates safer long-term administration and effective transduction of both dividing and non-dividing cells, rendering them appealing for targeting liver, hematopoietic stem cells, and endothelial cells (135). Research has shown effective hepatic endothelial cell transduction with pseudotyped LVs, reaching 95% GFP expression while maintaining angiogenic function (136). Targeting approaches like the blockade of Kupffer cells using gadolinium chloride have enhanced hepatocyte transduction (135, 136). In contrast to conventional techniques such as electroporation, LVs demonstrated enhanced gene transfer efficiency in liver sinusoidal endothelial cells (122). Additional improvements, such as microRNA-controlled constructs for liver cell-specific expression, have improved safety profiles (137, 138), yet systemic delivery issues remain (139). In HA, LVs that provide optimized FVIII through hematopoietic stem cells or platelets effectively regained clotting ability in animal models (140, 141). Comparable progress has been shown in HB models applying hepatocyte-targeted or platelet-directed lentiviral methods (133, 142).

In spite of these promising advancements, numerous translational obstacles must be surmounted prior to the clinical application of lentiviral methodologies (133). Specifically, the production of vectors on a large scale remains intricate and expensive, particularly when juxtaposed with AAVs, thereby constraining broader accessibility and scalability. Furthermore, the availability of long-term safety data in human subjects is scant, particularly with respect to clonal expansion or possible oncogenic occurrences post-integration, which necessitates stringent monitoring protocols in clinical investigations (134). Another pivotal difficulty resides in achieving targeted tissue delivery without eliciting immune recognition, a challenge that may be mitigated through the employment of innovative pseudotyping envelopes or specialized nanoparticles to enhance tissue tropism while safeguarding vectors from neutralizing antibodies (135). Lastly, although LV-based hematopoietic stem cell treatments have demonstrated sustained expression in animal models of hemophilia, their transition to clinical use requires optimized conditioning strategies that reduce toxicity while facilitating effective engraftment (136). Addressing these hurdles will be crucial for converting the biological potential of lentiviral vectors into safe, efficient, and scalable gene therapies for hemophilia and other monogenic conditions.

### 7.3 CRISPR-Cas-based gene editing strategies for hemophilia

Clustered regularly interspaced short palindromic repeats (CRISPR) along with CRISPR-associated (Cas) proteins have transformed gene therapy, especially for single-gene conditions like hemophilia. A key benefit of the CRISPR-Cas9 system is its accuracy and adaptability for fixing disease-related mutations at the genetic level. Huai et al. showed that *in vivo* hydrodynamic tail vein injections of CRISPR-Cas9 components combined with donor DNA effectively corrected the *F9* mutation in a mouse model of HB, restoring coagulation function in about 62.5% of the treated mice. *In vivo* microinjection of different Cas9 variants into germline cells further emphasized the safety and effectiveness of this method (143–146). Further research has concentrated on the albumin gene locus as a secure site for the insertion of therapeutic genes. Chen et al. documented effective homology-independent integration of the FIX gene at the albumin locus in a rat model, eliminating the requirement for exact homology-directed repair (142). Nonetheless, worries remain about AAV-mediated delivery in CRISPR applications, since AAV genomes may integrate into double-strand breaks caused by CRISPR. To tackle this issue, Breton et al. created ITR-Seq, a next-generation sequencing method that maps AAV integrations *in vivo*, providing enhanced understanding of genome editing safety (145).

To reduce extended Cas9 activity and its related risks, various research teams have investigated temporary delivery approaches. Intellia Therapeutics, for instance, employed lipid nanoparticles (LNPs) along with AAV to transport CRISPR-Cas9 mRNA, a guide RNA aimed at the albumin gene, and a FIX cDNA donor template. This LNP-AAV hybrid approach allowed temporary Cas9 expression while maintaining stable FIX expression regulated by the native albumin promoter, thereby effectively lowering the risk of off-target effects and prolonged immune activation (146). In a similar manner, another research utilized a dual-delivery system that focused on the antithrombin gene using CRISPR-Cas9 through LNPs, while delivering human FIX via AAV-transported donor DNA, presenting a unique strategy for reinstating hemostasis in HB models (146). Even with these developments, difficulties persist. Continuous expression of bacterial Cas9 proteins, regardless of being delivered by AAV or LNPs, may provoke immune reactions that destroy modified cells and undermine therapeutic results (147). Consequently, “hit-and-run” methods, which include temporary genome editing without extended effects, are essential. Future pursuits encompass creating safer non-viral delivery systems, enhancing immune evasion strategies, and progressing editing technologies like base and prime editors to facilitate long-lasting, one-time cures for hemophilia and various genetic disorders (145–148).

## 8 Challenges and future directions in the global implementation of innovative hemophilia therapies

One of the principal challenges associated with the management of hemophilia is the inconsistency in access to newly established therapeutic interventions, which is affected by both healthcare policy and systemic limitations (149). For instance, a particular survey aimed at assessing access patterns revealed that 77.3% (*N* = 119) of healthcare professionals (HCPs) perceived equal access for both adults and children, whereas 8% reported enhanced access for adults and 11% for children. Variability was also noticeable in the availability of specific therapeutic modalities. Specifically, 64% of respondents indicated that access to innovative treatment alternatives, such as extended half-life (EHL) FVIII, FIX, non-factor therapies including emicizumab, and gene therapy, was confined to specific clinical indications. Country-specific evaluations illustrated that such access limitations were acknowledged by 91% (*N* = 20/22) of participants in Canada, 63% (*N* = 17/27) in Italy, and 100% (*N* = 6/6) in New Zealand. Product-specific access restrictions were recognized by 35% (*N* = 54) of respondents concerning EHL-FVIII, 31% (*N* = 47) for EHL-FIX, 49% (*N* = 76) for non-factor therapies, and 48% (*N* = 74) for gene therapy (150). These results underscore the lack of universal access and suggest that the frameworks of healthcare systems and policy determinations may favor certain patient subpopulations, ultimately resulting in disparate treatment outcomes among individuals with analogous clinical requirements.

Furthermore, significant cross-national disparities persist in the availability of advanced hemophilia treatments, even among nations classified as high-income and upper-middle-income (150). This observed variability seems to be shaped by variations in the structures of healthcare systems, funding methodologies, and policy priorities. Nations characterized by government-funded universal healthcare exhibited distinct patterns in comparison to those employing fragmented multi-payer models, such as that of the United States. Intra-national regional disparities were also apparent, influenced by administrative protocols, fiscal allocations, and procurement strategies. Numerous healthcare professionals (HCPs) expressed a deficiency in understanding the healthcare organization and decision-making frameworks that govern access to innovative therapies, potentially diminishing their capacity to advocate effectively for patients. Proposed interventions encompassed specialized training initiatives, interprofessional communication platforms, and international knowledge-sharing endeavors to enhance collaboration among clinicians, payers, health technology assessment (HTA) entities, and policymakers, with the overarching objective of ensuring equitable and sustainable access (150, 151).

Another salient challenge pertains to the deficiency of transparency in the methodologies and procedural frameworks employed by national Health Technology Assessment (HTA) entities or analogous institutions in the evaluation of reimbursement eligibility for hemophilia therapies. Notably, awareness of an HTA authority was acknowledged by 90% (*N* = 109/121) of healthcare professionals (HCPs); however, 8.41% (*N* = 9/107) expressed that the evaluative process was characterized by a lack of clarity and transparency, while an additional 12.15% (*N* = 13/107) remained uncertain regarding the existence of a transparent process. The predominant evaluative methodologies encompassed cost-effectiveness analysis (61.7%) and budget impact analysis (38.9%). Perceptions of inconsistency or inadequate transparency in these assessments may undermine trust and hinder the incorporation of innovative therapies into established treatment protocols. Furthermore, a pronounced dependence on randomized controlled trials, which are considered the most critical source of evidence by 49.3% of participants, places treatments for rare diseases at a disadvantage, as extensive studies are frequently impractical. Consequently, there is an imperative need for more transparent and inclusive HTA processes to harmonize the expectations of payers, clinicians, and patients, thereby facilitating the acknowledgment of diverse evidence types (150).

Furthermore, there exists a limited formal influence exerted by patient organizations on reimbursement determinations for hemophilia therapies. Although a substantial majority of respondents (97.4%, *N* = 150) affirmed the presence of such organizations within their respective countries and 79.9% (*N* = 123) reported that these groups actively advocate for enhanced access, only 18.8% (*N* = 29) indicated that they possess formal voting rights in funding decisions related to approved therapies (150). This disparity between advocacy initiatives and actual decision-making power restricts the incorporation of patient perspectives, lived experiences, and considerations regarding quality of life into reimbursement frameworks (152). Structural reforms that ensure the substantial participation of patient organizations in formal decision-making processes could promote patient-centeredness, enhance adherence, and optimize long-term treatment outcomes.

Economic factors represent a major determinant influencing the availability and utilization of advanced hemophilia treatments in clinical practice. Survey data indicate that 40% of healthcare professionals (HCPs) acknowledged situations where therapeutic decisions are influenced by financial considerations, and 44.8% reported that the cost of hemostatic agents outside clinical trials significantly impacts patient access. Cost-related access limitations were most prevalent in New Zealand (66.7%, *N* = 4/6), England (62.5%, *N* = 10/16), and the USA (60.6%, *N* = 20/33). High development and manufacturing costs associated with newer therapeutic modalities, which are often substantially more expensive than standard factor replacement therapy, contribute to these challenges (150). Gene therapy and non-factor biologics require complex production systems, significant research investment, and strict regulatory compliance, resulting in elevated market prices. Even in high-income countries, payers may implement restrictive reimbursement policies or impose administrative barriers to control healthcare expenditure (152). Such financial constraints can delay or limit adoption of clinically beneficial treatments and can create ethical dilemmas when clinicians must balance optimal patient care with institutional budgetary restrictions (151). These findings underscore the importance of implementing comprehensive health economic analyses, including cost-effectiveness and budget impact assessments, to support transparent pricing negotiations and to promote broader and more equitable access to innovative therapies.

High treatment costs and the challenges of generating robust cost-effectiveness evidence remain significant barriers in hemophilia management (150). HA and HB are rare X-linked bleeding disorders with estimated incidences of approximately 1 in 5,000–10,000 and 1 in 25,000 male births respectively, and both require lifelong factor replacement therapy that accounts for over 90% of direct medical costs. Prophylactic regimens, now the standard of care for severe disease due to their proven benefits in joint protection, pain reduction, and improved quality of life, are considerably more expensive than on-demand treatment. In the United States, the average annual direct cost per patient without inhibitors is estimated at $185,256 (2011 USD), whereas patients with inhibitors face costs exceeding $400,000, and immune tolerance induction (ITI) therapy may range from $1 million to over $4 million depending on prognosis (2,000 USD). Inhibitor development, affecting up to one-third of severe hemophilia A patients and about 5% of severe hemophilia B cases, further complicates treatment and increases costs. The rarity of the condition, combined with heterogeneity in patient populations, limits the feasibility of large-scale cost-utility analyses and complicates long-term quality-adjusted life year (QALY) projections (153). Differences in orphan disease definitions across countries, along with the lack of effective treatments for many rare conditions, add to the difficulty of producing generalizable cost-effectiveness data. These constraints contribute to uncertainty in resource allocation decisions and highlight the need for tailored economic evaluation strategies that reflect the clinical and societal value of hemophilia therapies.

Hemophilia management in resource-limited nations (RLN) is significantly affected by a combination of high treatment costs, low diagnosis rates, and fragile healthcare infrastructure. As clotting factor concentrates account for more than 90% of total care costs, their accessibility and continuous supply remain critical. Although RLN host approximately 80% of the global hemophilia population, only 10–20% of patients are diagnosed, indicating the need for targeted diagnostic expansion alongside treatment provision. Effective strategies related to hemophilia care in these settings include the development of patient advocacy groups, establishment of national registries, and allocation of dedicated government budgets informed by diagnosed case numbers and scalable projections (154). Optimization of concentrate usage through appropriate dosing, prevention of wastage by improving supply chain management, and the use of near-expiry yet effective products can extend limited budgets. Plasma-derived intermediate purity factors and early-generation recombinant products, while less advanced, remain safe and affordable options. Long-term sustainability in RLN is related to investment in self-sufficiency through domestic plasma-based product manufacturing and the potential engagement of international companies to produce cost-effectively within national borders. Primary prophylaxis at low-to-intermediate doses can reduce inhibitor development and improve outcomes, while adherence will become increasingly important as access improves. Integration of non-factor therapies and participation in global procurement mechanisms can further strengthen care systems, supporting the transition from reactive, crisis-driven management to a sustainable, preventative framework (154–156).

One of the foremost impediments associated with the management of hemophilia in developing nations is the inadequacy of healthcare infrastructure and human resources specifically designed to cater to the requirements of persons with hemophilia (PWH). The provision of comprehensive care necessitates the formation of coordinated multidisciplinary teams that encompass hematologists, specialized nursing staff, physiotherapists, coagulation laboratory technicians, and access to diagnostic and therapeutic facilities. Numerous countries lack such cohesive systems. The emigration of skilled healthcare practitioners to higher-income countries exacerbates the domestic shortages, while the internal migration from rural areas to urban centers concentrates expertise within select hubs, thereby leaving substantial rural populations underserved. Typically, hospitals, laboratories, and rehabilitation facilities are situated in metropolitan regions, which creates obstacles to consistent access for rural PWH. Although private healthcare may offer enhanced facilities, the financial burdens are often prohibitive for the majority of patients (157). This inequitable distribution of resources directly constrains early diagnosis, timely intervention, and the long-term management of the disease. The insufficiency of trained multidisciplinary teams also hinders the implementation of evidence-based protocols, thus diminishing the efficacy of available resources. In the absence of strategic investments in human capital, infrastructure, and retention policies, sustainable advancements in hemophilia care within developing nations will remain challenging to realize (158, 159).

Limited access to medical insurance schemes constitutes another significant obstacle concerning hemophilia care in low-resource contexts. In numerous developing nations, health expenditure constitutes only 1–2% of GDP, with government funding for high-cost, low-prevalence disorders such as hemophilia being exceedingly minimal. This situation compels families to incur substantial out-of-pocket expenses, necessitating difficult decisions between substandard care and financial distress (157). Unlike high-income nations that possess comprehensive national health systems, public insurance schemes in these environments are frequently fragmented or entirely absent. Private insurance rarely encompasses genetic disorders, and when it is available, premiums are often unaffordable for the majority of the population. This financial gap contributes to delays in diagnoses, diminished frequency of treatment, and reliance on less effective alternatives such as fresh frozen plasma or cryoprecipitate. Preventive measures like prophylaxis, although cost-effective in the long run, necessitate upfront investments that are unfeasible for most households. Policymakers frequently prioritize conditions with a higher burden over rare bleeding disorders, thereby perpetuating budgetary neglect. Addressing these disparities will require the development of innovative insurance models, targeted governmental subsidies, and public-private partnerships to guarantee consistent and equitable access to life-saving therapies (158).

Competing health priorities constitute a significant obstacle in the management of hemophilia within developing nations. These nations grapple with the dual burden of communicable diseases, such as malaria, tuberculosis, and hepatitis, in conjunction with an increasing incidence of non-communicable diseases, which include diabetes, cardiovascular disorders, and malignancies. Budgetary constraints necessitate that governments direct their resources toward conditions exhibiting higher prevalence rates, thereby diminishing hemophilia’s status as a funding priority. The insufficient visibility of persons with hemophilia (PWH) within health care systems, resulting from underdiagnosis, the lack of comprehensive registries, and elevated mortality rates among undiagnosed individuals, further contributes to the marginalization of this condition (157). Historical misconceptions regarding the prevalence of hemophilia in specific regions have only recently been addressed through advancements in diagnostic technologies, thereby uncovering previously unrecognized patient populations. In the absence of comprehensive epidemiological data and effective advocacy efforts, hemophilia continues to be underrepresented in policy formulation, thereby obstructing the establishment of specialized care centers, access to factor concentrates, and the implementation of prophylactic programs. It is essential to enhance national data systems and advocacy initiatives to elevate hemophilia as a recognized public health concern and to secure adequate investment in care delivery. The inconsistent availability of factor concentrates poses a persistent operational challenge concerning hemophilia care in developing countries (159). The World Federation of Hemophilia recommends a minimum provision of 2 International Units per person annually; however, numerous countries do not meet this standard, with the majority of patients relying on less effective alternatives. Even when concentrates are accessible, the associated costs remain exorbitant, as on-demand therapy for a 50 kg adult can reach $3,000–4,000 USD annually, an amount insufficient to avert chronic joint deterioration. Inefficiencies within supply chains, bureaucratic procurement processes, and inadequate distribution systems exacerbate shortages and resultant wastage. An over-reliance on humanitarian assistance renders health systems susceptible to supply interruptions. Inequities in the distribution of stock among facilities further restrict equitable access. Domestic plasma fractionation initiatives, as exemplified by Brazil, present a viable pathway toward enhanced self-sufficiency; however, such initiatives necessitate robust political commitment, regulatory adaptability, and significant infrastructural investment. Strategic planning for sustainable production and procurement is crucial to rectify chronic under-supply and to enhance long-term patient outcomes (157, 160).

A multitude of clinical management challenges is also associated with hemophilia treatment in developing nations, including inadequate utilization of adjunct therapies, limited options for pain management, elevated viral infection rates, and the development of inhibitors. Supportive interventions such as physiotherapy, antifibrinolytic agents, orthotics, and tailored surgical planning are frequently underutilized due to insufficient training and poor allocation of resources. Options for pain management are often confined to paracetamol or sporadic usage of COX-2 inhibitors, with opioids being either unavailable or subject to stringent regulation, resulting in inadequate management of chronic pain. Insufficiently screened blood products contribute to elevated prevalence rates of hepatitis B and C among PWH, thereby increasing morbidity and complicating long-term care strategies. The development of inhibitors remains a significant therapeutic obstacle, particularly in light of restricted access to bypassing agents and immune tolerance induction therapies. The minimal output of local research further constrains the adaptation of international best practices to the specific contexts of these nations. Enhancing care will necessitate comprehensive strategies that encompass infection control, pain management, rehabilitation, and the expansion of context-specific research networks (157–160).

One of the major challenges related to hemophilia care is ensuring equitable access, coordinated management, and sustainable models of service delivery across different healthcare settings. Disparities in healthcare infrastructure and resource allocation between countries result in unequal access to innovative therapies and specialized hemophilia treatment centers (HTCs). While the administration of factor concentrates is acknowledged as the prevailing method for managing severe hemophilia, the substantial financial implications associated with these interventions, including advanced formulations with extended half-lives and innovative therapies such as emicizumab, significantly constrain their accessibility in resource-limited environments (161). This economic obstacle similarly impacts the feasibility of ITI therapy for patients with inhibitors, wherein treatment expenses may surpass USD 1 million, thereby hindering equitable access. The infrequency of the condition further exacerbates the challenges associated with establishing standardized care guidelines, whereas the heterogeneous requirements of patients demand a multidisciplinary approach that may prove challenging to uphold within financially constrained healthcare systems. The scarcity of training prospects for healthcare practitioners, especially in the domains of physical therapy, psychological support, and dental services, compounds the challenges faced in delivering comprehensive and integrated healthcare solutions (109).

Another significant challenge associated with the management of hemophilia pertains to the care of carriers and the incorporation of psychosocial support within treatment frameworks. Numerous female carriers exhibit clinically significant bleeding attributed to diminished coagulation factor levels; however, the processes for identification and counseling remain markedly inadequate (152). Although genetic testing has advanced technologically, its implementation is inconsistent, resulting in many carriers lacking awareness of their status, reproductive options, and related risks. The integration of mental health care for both patients and their families is sporadically included, despite its evident importance for enhancing quality-of-life outcomes. The transition from pediatric to adult healthcare represents a particularly vulnerable phase characterized by risks of treatment discontinuity, diminished adherence, and increased psychosocial stress. Continuous interprofessional collaboration, comprehensive patient education, and supportive health policies are imperative; nevertheless, such initiatives are frequently obstructed by workforce shortages, fragmented inter-disciplinary communication, and insufficient funding (157).

A further array of challenges concerning hemophilia treatment pertains to the execution of gene therapy, which encompasses a multitude of technical, biological, ethical, financial, and social factors. Limitations associated with viral vectors are notably pronounced, as pre-existing anti-AAV antibodies are detected in 30–80% of individuals, thereby diminishing transduction efficiency and excluding numerous candidates from clinical trials. Potential remedial strategies, such as utilizing alternative serotypes like AAV5 in the presence of anti-AAV5 antibodies or employing antibody degradation techniques, including imlifidase, remain in the experimental phase. Hepatotoxicity manifests in approximately 60% of recipients within a timeframe of 4–12 weeks, frequently necessitating the administration of corticosteroid therapy (109, 157). Although AAV vectors do not integrate into the host genome, the potential for integration events and the occurrence of hepatocellular carcinoma observed in preclinical studies necessitate ongoing surveillance. Rare instances of germline transmission documented in preclinical investigations support current guidelines advocating for post-treatment contraception. The durability of therapy exhibits variability, with FIX expression maintained for a minimum of 5 years in certain cases, while FVIII expression often diminishes within a four-year period. Ethical dilemmas emerge in pediatric applications due to vector loss during hepatic growth, and in particular cultural contexts, such as Muslim-majority societies, where acceptance may hinge on principles of public benefit (99, 109, 162).

Financial limitations persist as a pivotal issue regarding the implementation of gene therapy for hemophilia. The cost of AMT-061, for instance, is approximately USD 3.5 million per dose. While cost-effectiveness analyses suggest potential savings in comparison to lifelong prophylaxis, the substantial initial expenditure constitutes a considerable barrier to adoption, particularly in low- and middle-income nations. Proposed payment models, such as outcome-based contracts, subscription frameworks, or volume-linked agreements, encounter pragmatic and definitional challenges. On a societal level, awareness among both patients and healthcare professionals remains limited, as surveys reveal that approximately one-third of physicians find it challenging to elucidate AAV-based therapy, and 40% express feeling unprepared to tackle patient inquiries. Eligibility criteria are additionally constrained by comorbid conditions such as HIV and hepatitis, the presence of inhibitors, and advanced patient age. Although post-marketing research may broaden inclusion criteria, issues related to pricing and infrastructural limitations endure, highlighting the necessity for equitable cost structures, robust delivery systems, and targeted educational initiatives (153, 157).

## 9 Conclusion

Gene therapy has represented a major advancement in hemophilia treatment, altering the clinical condition of numerous patients suffering from severe forms into milder types by bringing clotting factor levels close to normal ranges. One intravenous dose of AAV-based gene therapies has shown the ability to greatly decrease or even stop spontaneous bleeding episodes, providing patients relief from the obligation of standard preventative treatments. Nonetheless, various challenges remain, including fluctuations in FVIII expression over time, immune-mediated responses, and hypersensitivity, which currently limit eligibility to only a subset of patients, particularly those without liver disease or pre-existing AAV antibodies.

An additional limitation is the challenge of forecasting and regulating immune reactions to AAV vectors. Reactions related to infusion, liver impairment, and worries about possible long-term toxicity, including the danger of cancers, highlight the necessity for vigilance. Immunogenicity continues to be a significant challenge, as both innate and adaptive immune responses hinder the effectiveness of gene delivery and complicate the re-administration of vectors. Recent clinical events have emphasized the necessity for stronger preclinical models and approaches to inhibit or circumvent immune activation. Methods like immune modulation, capsid modification, and targeted delivery are being investigated to reduce these hazards. Additionally, figuring out the best dosing regimens remains a complicated challenge. Although underdosing can result in inadequate therapeutic effectiveness, excessive doses increase the likelihood of toxicity and unintended effects. Allometric scaling models have been developed to aid in the selection of initial patient doses, with certain models showing greater reliability than others. Furthermore, innovative techniques such as plasmapheresis and immune suppression are undergoing evaluation to enable repeated AAV treatments in patients with antibodies. Experimental methods utilizing IgG-degrading enzymes and modulation of T-cell pathways have demonstrated potential in enhancing transduction rates during vector reintroduction.

However, advancing hemophilia gene therapy requires not only overcoming immunological barriers but also addressing disease-specific gaps, particularly in HB. This condition remains underrepresented in translational research and clinical trials. Integrating HB-specific therapeutic approaches, mutation-guided patient stratification, and the inclusion of symptomatic female carriers into research frameworks is vital for comprehensive innovation.

Equally important is ensuring that these scientific advances reach all populations globally. Current treatment disparities between high-income and LMICs, including limited access to factor replacement and exclusion from clinical trials, demand targeted strategies. Sustainable pricing models, international aid programs, regional infrastructure for gene therapy and HSC transplantation, and investments in physician education and telemedicine must be scaled to reduce inequity.

In the future, the subsequent generation of gene therapy for hemophilia will probably include more tailored strategies based on personal immune profiles and genetic histories. Advancements in vector design, alternative delivery methods, and affordable manufacturing techniques will be crucial in expanding accessibility and guaranteeing safety. The primary objective continues to be the creation of safe, effective, and long-lasting gene therapies that offer lifelong advantages with minimal intervention, transforming hemophilia into a manageable condition for various patient groups and healthcare frameworks.
